# Protein and metabolic profiles of tyrosine kinase inhibitors co-resistant liver cancer cells

**DOI:** 10.3389/fphar.2024.1394241

**Published:** 2024-05-21

**Authors:** Zengbin Wang, Linqing Wu, Yu Zhou, Zhong Chen, Tao Zhang, Hong Wei, Zhihong Wang

**Affiliations:** ^1^ Department of Immunology, School of Basic Medical Sciences, Fujian Medical University, Fuzhou, China; ^2^ Department of Clinical Pharmacy and Pharmacy Administration, School of Pharmacy, Fujian Medical University, Fuzhou, China; ^3^ Department of Hepatobiliary Surgery, Fujian Provincial Hospital, Fuzhou, China; ^4^ Shengli Clinical Medical College of Fujian Medical University, Fuzhou, China; ^5^ Department of Cadres’s Healthcare Office, Fujian Provincial Hospital, Fuzhou, China; ^6^ Department of Hematology, Fujian Provincial Hospital, Fuzhou, China

**Keywords:** sorafenib, lenvatinib, drug resistance, proteomics, metabolomics, hepatocellular carcinoma

## Abstract

Hepatocellular Carcinoma (HCC) patients often develop resistance to tyrosine kinase inhibitors (TKIs) like sorafenib (SR) and lenvatinib (RR). We established HCC cell lines resistant to these drugs and analyzed the correlation between protein and metabolite profiles using bioinformatics. Our analysis revealed overexpression of MISP, CHMP2B, IL-18, TMSB4X, and EFEMP1, and downregulation of IFITM3, CA4, AGR2, and SLC51B in drug-resistant cells. Differential signals are mainly enriched in steroid hormone biosynthesis, cell adhesion, and immune synapses, with metabolic pathways including cytochrome P450 drug metabolism, amino acid metabolism, and glycolysis. Proteomics and metabolomics analysis showed co-enrichment signals in drug metabolism, amino acids, glucose metabolism, ferroptosis, and other biological processes. Knocking down MISP, CHMP2B, IL-18, TMSB4X, and EFEMP1 significantly reduced drug resistance, indicating their potential as therapeutic response biomarkers. This study characterizes protein and metabolic profiles of drug-resistant HCC cells, exploring metabolite-protein relationships to enhance understanding of drug resistance mechanisms and clinical treatment.

## Introduction

Sorafenib and lenvatinib, both belonging to the class of tyrosine kinase inhibitors (TKIs), serve as molecularly targeted drugs for the treatment of HCC. Their primary focus is on suppressing multiple crucial targets involved in tumor angiogenesis, signal transduction pathways, and immune regulation (Chan and Chan, 2023; Villarruel-Melquiades et al., 2023). Sorafenib and lenvatinib effectively restrain the advancement of HCC by impeding an assortment of receptor tyrosine kinases (RTKs) (Faivre et al., 2020). Sorafenib primarily suppresses the growth and angiogenesis of HCC cells by targeting and inhibiting vascular endothelial growth factor receptors (VEGFR) as well as the Raf/MEK/ERK signaling pathway (Jiang et al., 2023). In addition, sorafenib can also affect the regulation of tumor related immune cells and their factors, promoting the body’s immune response. Lenvatinib mainly inhibits various receptors and pathways within cells, such as VEGFR, platelet-derived growth factor receptors (PDGF), fibroblast growth factor receptors (FGFR) and hepatocyte factor receptor (c-KIT), to inhibit cell proliferation, promote cell apoptosis, and block angiogenesis, thereby achieving the effect of treating HCC (Laface et al., 2022). Despite the availability of TKIs, the reality is that most patients with advanced HCC eventually develop either innate or acquired resistance to these therapies (De Mattia et al., 2019). It is imperative to develop novel treatment strategies to address TKI resistance in advanced HCC patients.

Recent studies have shown that epigenetics, transport processes, regulated cell death, tumor microenvironment, hypoxia and viral reactivation play a role in the production and development of sorafenib resistance in HCC (Tang et al., 2020; Ladd et al., 2024). Lenvatinib resistance also has a similar mechanism to sorafenib resistance, including noncoding RNA regulation, tumor immune microenvironment and expansion of cancer stem cells (Tao et al., 2023). In addition, the combination of immune checkpoint inhibitors (ICI) and TKIs has important therapeutic significance in the treatment of HCC (Starzer et al., 2024). However, understanding the mechanism of TKIs resistance from a global perspective is still unclear.

The aim of this study is to investigate the mechanism of TKIs resistance and identify new therapeutic targets. In addition, this study attempts to determine the protein and metabolic profiles of TKIs resistance, providing insights for overall treatment rather than targeted therapy alone.

## Materials and methods

### Reagents and kits

Reagents and antibodies are as follows: sorafenib (MedChemExpress, Cat# HY-10201); lenvatinib (MedChemExpress, Cat# HY-10981); fetal bovine serum (Gemini Bio, Sacramento, CA); Lipofectamine 3,000 (Invitrogen); Dulbecco’s Modified Eagle Medium (DMEM) (Gibco, Grand Island, NY); Fluoromount with 4′, 6- diamidino-2-phenylindole (DAPI) (Sigma-Aldrich, St. Louis, MO); Puromycin (Solarbio Life Science, Beijing, China); GAPDH (Proteintech, Cat# 60004-1-Ig); MISP (Proteintech, Cat# 26338-1-AP); CHMP2B (Proteintech, Cat# 12527-1-AP); TMSB4X (Proteintech, Cat# 19850-1-AP); IL-18 (ABclonal, Cat# A1115); EFEMP1 (Abcam, Cat# ab256457).

### Clinical specimens

Retrospective collection of tissue paraffin embedded samples from HCC patients admitted to Fujian Provincial Hospital from January 2023 to December 2023. This research protocol has been approved by the Ethics Committee of Fujian Provincial Hospital (K2023-05-016).

### Cell lines

The Huh7 cell lines (RRID: CVCL_0336, JCRB0403, Japan) were cultured in DMEM, supplemented with 10% FBS and 100 U/mL Penicillin/Streptomycin, and maintained at 37°C in a 5% CO_2_ atmosphere. The cells underwent routine testing for *mycoplasma* contamination, which confirmed their freedom from contamination.

### Establishment of sorafenib-resistant and lenvatinib-resistant HCC cells

The Huh7/SR and Huh7/RR cell lines, which are resistant to sorafenib or lenvatinib, were created in a previous study (Wang et al., 2023b; Leung et al., 2023). Briefly, Huh7 cells were cultured with gradually increasing doses of sorafenib or lenvatinib. Both Huh7/SR and Huh7/RR cells were cultured at a constant concentration of 10 μM.

### siRNA transfection

si *MISP* (target sequence: TTC​CGT​TTC​TAT​CTT​CCT​TTA​GA), si *CHMP2B* (target sequence: AAG​AAA​ACC​GTG​GAT​GGA​ATT​AG), si *IL-18* (target sequence: AAC​TAT​TTG​TCG​CAG​GAA​TAA​AG), si *TMSB4X* (target sequence: TAG​CTG​TTT​AAC​TTT​GTA​AGA​TG), si *EFEMP1* (target sequence: CGC​ACA​GAT​TCA​CAA​TGT​TGA​AA) and scrambled control si RNA (si Control), were purchased from Tsingke Biotechnology Co., Ltd. All siRNA transfections were performed using Lipofectamine 3,000 according to the manufacturer’s protocol.

### Cell viability assay

The CCK-8 reagent was used to test cell viability in accordance with the manufacturer’s instructions. In brief, the 96 well plate had 5,000 cells per well. Next, 10 μL of CCK-8 reagent was added to each well. The absorbance of every well was measured at 450 nm with a microplate reader (BioTek, Winooski, VT).

### Immunoblotting

Immunoblotting was based on our previous research (Wang et al., 2023a). The total protein samples (25 μg) were separated through SDS-PAGE were separated via SDS-PAGE and transferred onto PVDF membranes (Biosharp, Hefei, China). Following this, the membranes were blocked with 5% bovine serum albumin (BSA) at room temperature for 1 h. The primary antibodies were applied to the membranes and incubated overnight at 4°C. After this step, secondary antibodies were added and incubated at room temperature for 1 h. The resulting bands were detected and visualized using a Hypersensitive ECL Chemiluminescence Kit (NcmECL Ultra, ABP Biosciences, Beltsville, MD, USA). The study utilized several primary antibodies: anti-MISP (1:1,000), anti-CHMP2B (1:1,000), anti-IL-18 (1:1,000), anti-TMSB4X (1:1,000), and anti-EFEMP1 (1:1,000), anti-GAPDH (1:5,000).

### Cell proliferation assay

The EdU Cell Proliferation Kit (Solarbio, China) was utilized to quantify cell proliferation as per the guidelines furnished by the manufacturer. Upon combining with a fluorescent azide, proliferating cells emitted a vibrant red fluorescence that was visualized under a fluorescent microscope.

### 4D-DIA quantitative proteomics

4D-DIA quantitative proteomics was detected and analyzed by Novogen Co., Ltd. The software used for integrating metabolomics and proteomics data were presented in Supplementary Table S1. The steps are as follows.

#### Protein extraction

The sample was treated with lysis buffer (8 M urea supplemented with 1 mM PMSF and 2 mM EDTA) and subjected to ultrasonic waves to break down the cells. Following this, the residual debris was eliminated by centrifuging the mixture at 15000 g and at a temperature of 4°C for a duration of 10 min. BCA protein quantitation assay was employed to deduce the total protein concentration.

#### Digestion and cleanup

For tryptic digestion, an identical quantity of proteins from each sample was employed. The supernatants were supplemented with 8 M urea (200 µL) and reduced using 10 mM DTT at 37°C for a period of 45 min, following which they were alkylated using 50 mM iodoacetamide (IAM) at room temperature for 15 min in a darkroom. The resulting mixture was precipitated by adding 4× volume of chilled acetone and incubating at −20°C for 2 h. After centrifuging, the protein precipitate was air-dried, and then resuspended in a solution of 200 µL of 25 mM ammonium bicarbonate along with 3 µL of trypsin (Promega). The mixture was allowed to undergo overnight digestion at 37°C. Next, the resulting peptides were purified using a C18 Cartridge. Afterward, the peptides were dried using a Vacuum Concentration Meter, concentrated by vacuum centrifugation and eventually redissolved in a solution of 0.1% (v/V) formic acid.

#### LC-MS/MS analysis

A nanoElute UHPLC (Bruker Daltonics, Germany) was utilized to perform liquid chromatography (LC). The reverse-phase C18 column, which was commercially available with an integrated CaptiveSpray Emitter, allowed for the separation of approximately 200 ng peptides at a flow rate of 0.3 μL/min for 40 min. The integrated Toaster column oven maintained the separation temperature at 50°C. The mobile phases used were A and B, with 0.1 vol.-% formic acid in water and 0.1% formic acid in ACN respectively. Over the initial 25 min, mobile phase B was increased from 2% to 22%, and then, over the subsequent 5 min, it was augmented to 35%, further progressing to 80% over a period of subsequent 5 min while being held at 80% for a further 5 min. The LC was linked online to a hybrid timsTOF Pro2 (Bruker Daltonics, Germany) via a CaptiveSpray nano-electrospray ion source. In order to identify the suitable acquisition windows for diaPASEF mode, the timsTOF Pro2 was initially managed in Data-Dependent Parallel Accumulation-Serial Fragmentation (PASEF) mode with 4 PASEF MS/MS frames in 1 complete frame. The capillary voltage of 1500 V was set, while the MS and MS/MS spectra were gathered from 100 to 1700 m/z. As for the ion mobility range (1/*K*0), 0.85–1.3 Vs/cm2 was employed.

#### Database search and quantification

DIA-NN (v1.8.1) was utilized to analyze the MS raw data using a library-free approach. The uniprot_proteomeUP000005640_human_20230504.fasta database (which amounted to 82492 sequences) was employed to develop a spectra library via deep learning algorithms of neural networks. The MBR option was employed to produce a spectral library from DIA data, which was then reanalyzed utilizing this library. The search results were subjected to FDR adjustments to less than 1% at both protein and precursor ion levels; the remaining identifications were implemented for further quantification analysis.

### Untargeted metabolomics

Untargeted metabolomics was detected and analyzed by Novogen Co., Ltd. The steps are as follows.

#### Cell samples class I

A 500 μL solution, containing the internal standard, was added to the cell sample, vortexed for 3 min, and subsequently subjected to a single freeze-thaw cycle consisting of placement in liquid nitrogen for 5 min followed by dry ice for 5 min, after which it was thawed on ice and vortexed for 2 min. A 300 μL supernatant was collected and stored at −20°C for 30 min. Afterward, it was centrifuged once again at 12000 rpm for 3 min, at 4°C. Following which, 200 μL aliquots of the supernatant were transferred for LC-MS analysis.

#### HPLC conditions

All of the samples were subjected to two LC/MS assays. One aliquot was analyzed using positive ion conditions, and was eluted from the T3 column (Waters ACQUITY Premier HSS T3 Column 1.8 µm, 2.1 mm * 100 mm), utilizing 0.1% formic acid in water as solvent A and 0.1% formic acid in acetonitrile as solvent B across the following gradient: 5%–20% within 2 min, followed by an increase to 60% in the subsequent 3 min, then an increase to 99% within 1 min, followed by a retention time of 1.5 min, thereafter returning to the initial 5% mobile phase B within 0.1 min, and a retention time of 2.4 min. The second aliquot was analyzed using negative ion conditions, utilizing the same elution gradient as the positive mode.

#### MS conditions (AB)

Data acquisition was performed using the information-dependent acquisition (IDA) mode, and the Analyst TF 1.7.1 Software (Sciex, Concord, ON, Canada) was used for this purpose. The TOF MS scan parameters were set at a mass range of 50–1,000 Da, with an accumulation time of 200 ms, and a dynamic background subtract was enabled. The product ion scan parameters were set at a mass range of 25–1,000 Da, with an accumulation time of 40 ms.

### Statistical analysis

The results are expressed as mean ± SD and conducted using GraphPad Prism V.8. For normally distributed data with homogeneous variance, use unpaired t-tests to compare two samples. The comparison between multiple groups was conducted using one-way analysis of variance. A *p*-value <0.05 is considered statistically significant.

## Results

### Quality evaluation of quantitative results between proteomic samples

**FIGURE 1:**
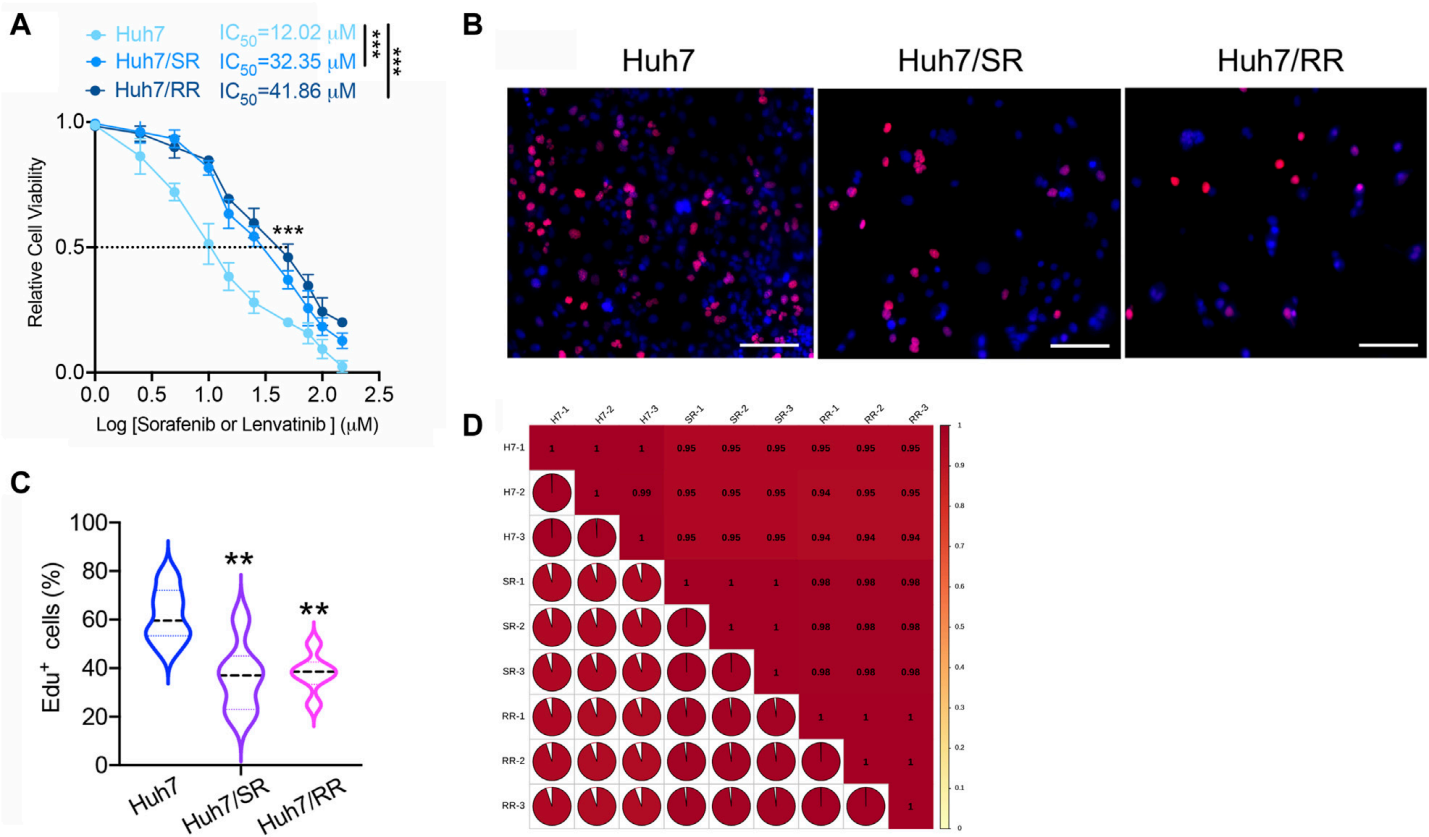
Quality evaluation of quantitative results between proteomic samples. **(A)** The IC_50_ values of Huh7, Huh7/SR, and Huh7/RR cells were detected. *n* = 3. Data were analyzed by unpaired *t*-test: ***p < 0.001. **(B,C)** The Edu proliferation detection kit was used to detect the number of Edu positive cells, *n* = 6. Bar = 50 μm. Data were analyzed using One-Way ANOVA analysis: **p < 0.01. **(D)** Pearson’s Correlation Coefficient. The value of |R| indicates the strength of the correlation between two samples, with values approaching 1 indicating a strong correlation.

We produced Huh7 sorafenib resistant and lenvatinib resistant cell lines (Huh7/SR, Huh7/RR), respectively. Compared to the parental Huh7 cells, both Huh7/SR and Huh7/RR cells have higher IC_50_ values (Figure 1A). We next used the EdU proliferation detection kit to compare the number of EdU positive cells between parental cells and drug-resistant cells. It was found that compared to parental cells, both sorafenib and lenvatinib resistant cells had fewer Edu positive cells (Figures 1B, C). This indicates a decrease in the proliferation ability of drug-resistant cells. To evaluate the co-resistance mechanism of these two types of drug-resistant cells, we used 4D-DIA quantitative proteomics technology to explore the differences in protein expression. Both Huh7 and Huh7/RR, as well as Huh7 and Huh7/SR, have correlation coefficients greater than or equal to 0.94 (Figure 1D).

### Protein differential expression analysis

**FIGURE 2:**
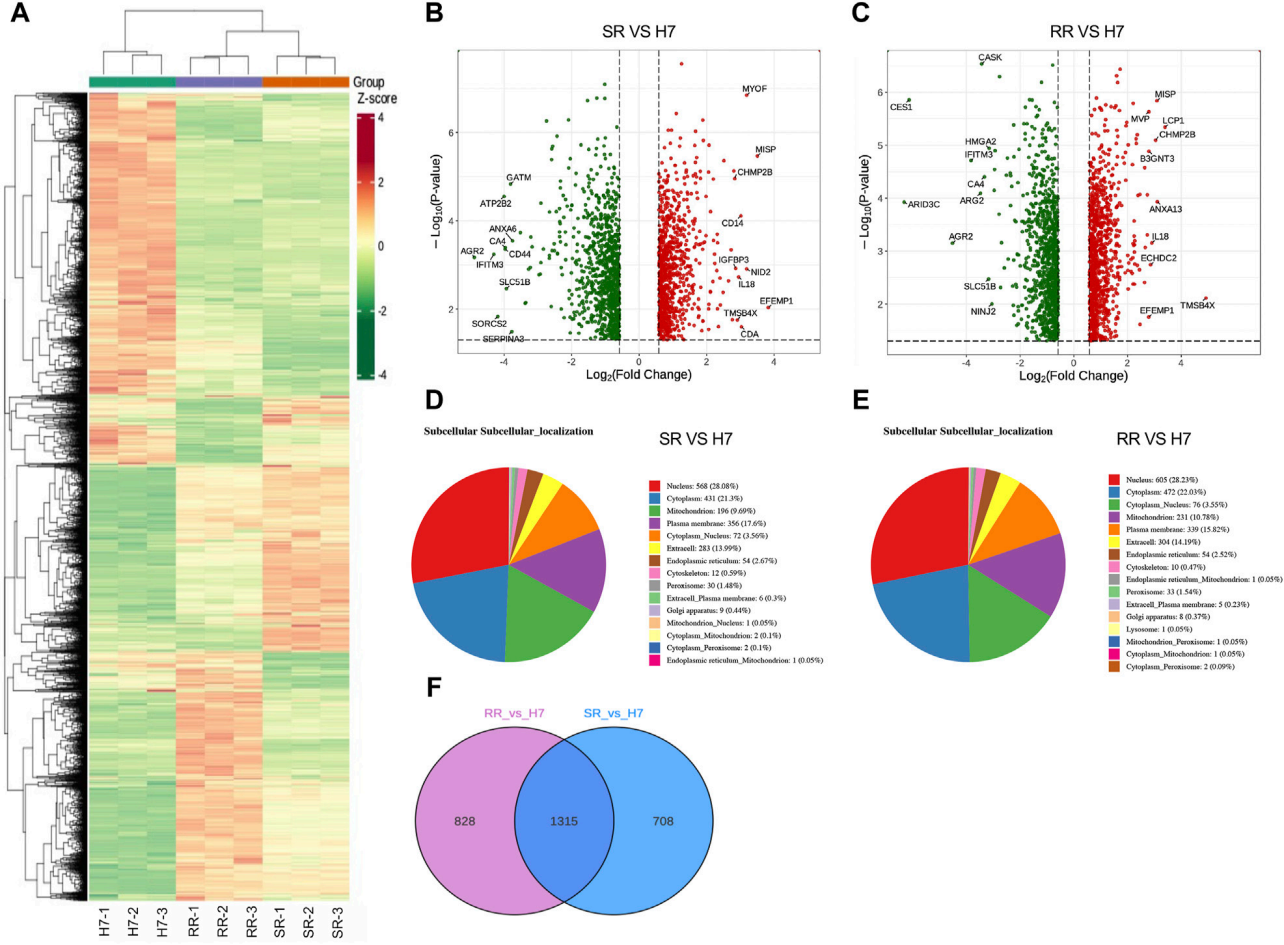
Protein differential expression analysis. **(A)** Differential protein clustering heatmap. Rows represent clustering of differentially expressed proteins, while columns represent clustering of samples. **(B,C)** Differential protein volcano plot. The horizontal axis represents log2 of the differential multiple, the vertical axis represents -log_10_ *p*-value, and the red and green scatter dots represent the up and downregulated differential proteins. **(D,E)** Subcellular localization of proteins that were differentially expressed is depicted with each subcellular compartment represented by a distinct color. The number of proteins that were annotated for each subcellular compartment is noted outside the parentheses, while the proportion of differentially expressed proteins annotated for each subcellular compartment is noted within the parentheses as compared to the total subcellular annotation. **(F)** Differential protein Venn diagram. *n* = 3.

Cluster heatmaps display differences in protein expression patterns between two drug-resistant cells and parental cells (Figure 2A). In comparison to Huh7 cells, Huh7/SR cells displayed an increase in 972 protein expressions and a decrease in 1,051 protein expressions. Similarly, Huh7/RR cells showed an increase in 1,071 protein expressions and a decrease in 1,072 protein expressions (Figures 2B, C). Compared to Huh7/SR cells, Huh7/RR cells upregulated 504 proteins and downregulated 389 proteins (Supplementary Figure S1). Notably, we identified the top 10 upregulated and downregulated proteins. Co-upregulated proteins included MISP, CHMP2B, IL-18, TMSB4X and EFEMP1, while co-downregulated proteins comprised of IFITM3, CA4, AGR2 and SLC51B (Figures 2B, C). Subcellular localization analysis of proteins found that they are mainly concentrated in the nucleus, cytoplasm, mitochondrion and plasma membrane (Figures 2D, E). Venn diagram showed that there are 1,315 common differentially expressed proteins in both groups (Figure 2F). These data indicate that many proteins in drug-resistant cells have undergone changes, indicating that the protein profile has been reconstructed.

### Functional enrichment of differentially expressed proteins

**FIGURE 3:**
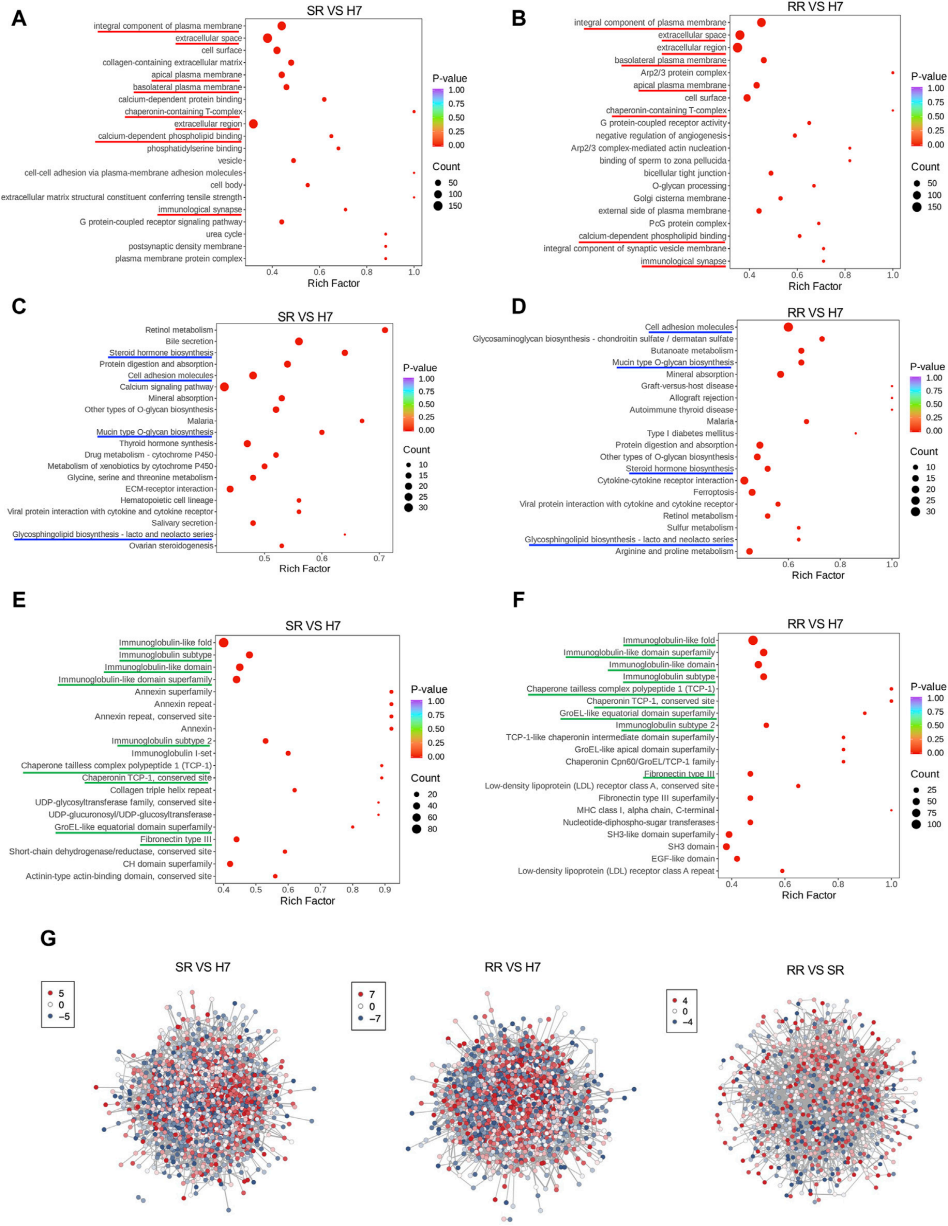
Functional enrichment of differentially expressed proteins. **(A,B)** Bubble charts depicted the GO enrichment analysis results. The horizontal axis showed the enrichment factor (DiffRatio/BgRatio ratio), reflecting the degree of enrichment, while the vertical axis displayed the name of the GO entry. **(C,D)** Bubble charts illustrated the KEGG enrichment analysis results. The horizontal axis showed the enrichment factor, reflecting the level of enrichment, while the vertical axis showed the KEGG pathway. **(E,F)** Bubble diagrams suggested the results of the structural domain enrichment analysis. The horizontal axis exhibited the enrichment factor, reflecting the level of enrichment, while the vertical axis showed the description of the IPR entry. **(G)** Differential expression protein interaction network. The differential expression protein interaction network diagram demonstrated the differentially expressed proteins. Each node represented a protein, with color change from red to blue indicating the expression level change from up to down. *n* = 3.

The gene ontology (GO) enrichment analysis revealed that, in comparison to Huh7 cells, both Huh7/SR and Huh7/RR cells exhibited shared differential enrichment signals. These signals were primarily associated with integral components of the plasma membrane, extracellular space, extracellular region, basolateral plasma membrane, apical plasma membrane, chaperonin-containing T-complex, calcium-dependent phospholipid binding, and immunological synapse (Figures 3A, B). KEGG pathway analysis showed that the common differential enrichment signals in Huh7/SR and Huh7/RR cells primarily involved steroid hormone biosynthesis, cell adhesion molecules, mucin type O-glycan biosynthesis, and glycosphingolipid biosynthesis—lacto and neolacto series (Figures 3C, D). Furthermore, the structural domain enrichment analysis revealed that the common differential structural domains in Huh7/SR and Huh7/RR cells were primarily associated with immunoglobulin-like folds and subtypes, immunoglobulin-like domains and their superfamily, immunoglobulin subtype 2, chaperone tailless complex polypeptide 1 (TCP-1), chaperonin TCP-1, conserved sites, groEL-like equatorial domain superfamily, and fibronectin type III (Figures 3E, F). Protein protein interaction analysis (PPI) showed that both Huh7/SR vs. Huh7 and Huh7/RR vs. Huh7 exhibited highly complex differentially expressed protein interactions, while the interaction of Huh7/RR vs. Huh7/SR was relatively reduced (Figure 3G). These results indicate that the Huh7/SR and Huh7/RR cell populations exhibit unique molecular characteristics, revealing rich pathways and structural domains involved in cellular signaling, biosynthesis, and immune responses, which can provide valuable insights for drug resistance in HCC treatment.

### Quality evaluation of quantitative results between metabolomic samples

**FIGURE 4:**
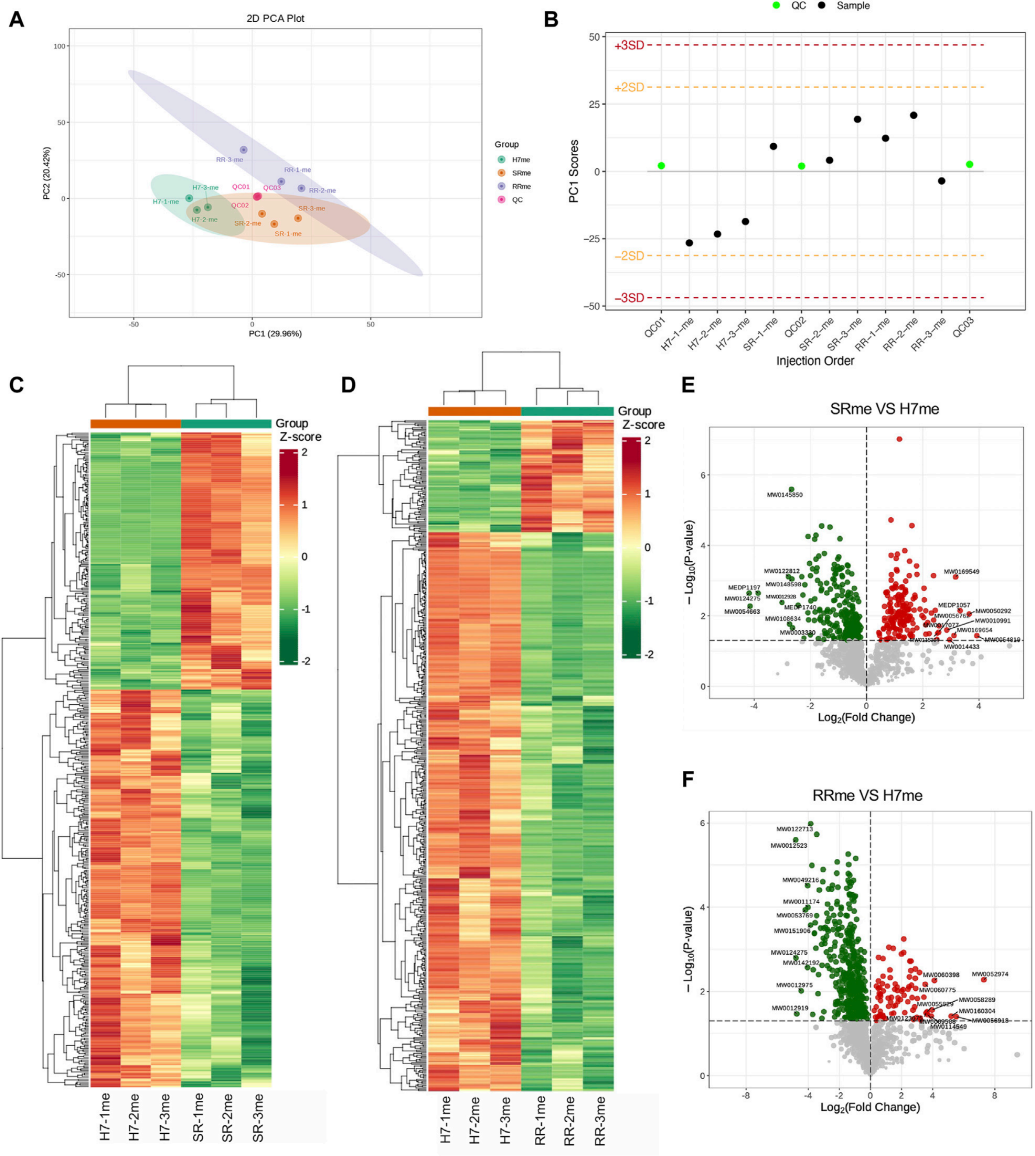
Quality evaluation of quantitative results between metabolomic samples. **(A)** PCA was performed to analyze the variance in the data. **(B)** The Sample PC1 control chart showed the PC1 value of the experimental and quality control samples plotted against the order of detection. The yellow and red lines depicted positive and negative 2 and 3 standard deviation ranges, respectively. **(C,D)** The cluster diagrams exhibited sample and metabolite information grouped according to the standardized relative content values. The horizontal axis represented sample information, the vertical axis represented metabolite information, and different colors indicated the degree of variation in the content (red represents high, green represents low). **(E,F)** Differential metabolite volcano map. *n* = 3.

Because both proteins and metabolites are closely related to cellular function. We have constructed a proteomic profile of drug-resistant cells, and we next continue to construct a metabolomic profile of drug-resistant cells. PCA results showed differences in metabolomic separation trends among groups (Figure 4A). PC1 Scores suggested that the test samples were within the range of 3 standard deviations (SD) (Figure 4B). The clustering heatmap provided the differences in metabolites between two types of drug-resistant cells and parental cells (Figures 4C, D). The differential metabolite volcano plots displayed a visual representation of the statistical significance and magnitude of differences in metabolite abundance between groups. Compared to the Huh7 cells, the Huh7/SR cells had 176 metabolites elevated and 272 metabolites decreased (Figure 4E), while the Huh7/RR cell group had 89 metabolites elevated and 444 metabolites decreased (Figure 4F). These results can aid in understanding the metabolic pathways and regulatory mechanisms underlying cellular function and disease, providing a basis for potential biomarker discovery and therapeutic intervention.

### Functional enrichment analysis of differential metabolites

**FIGURE 5:**
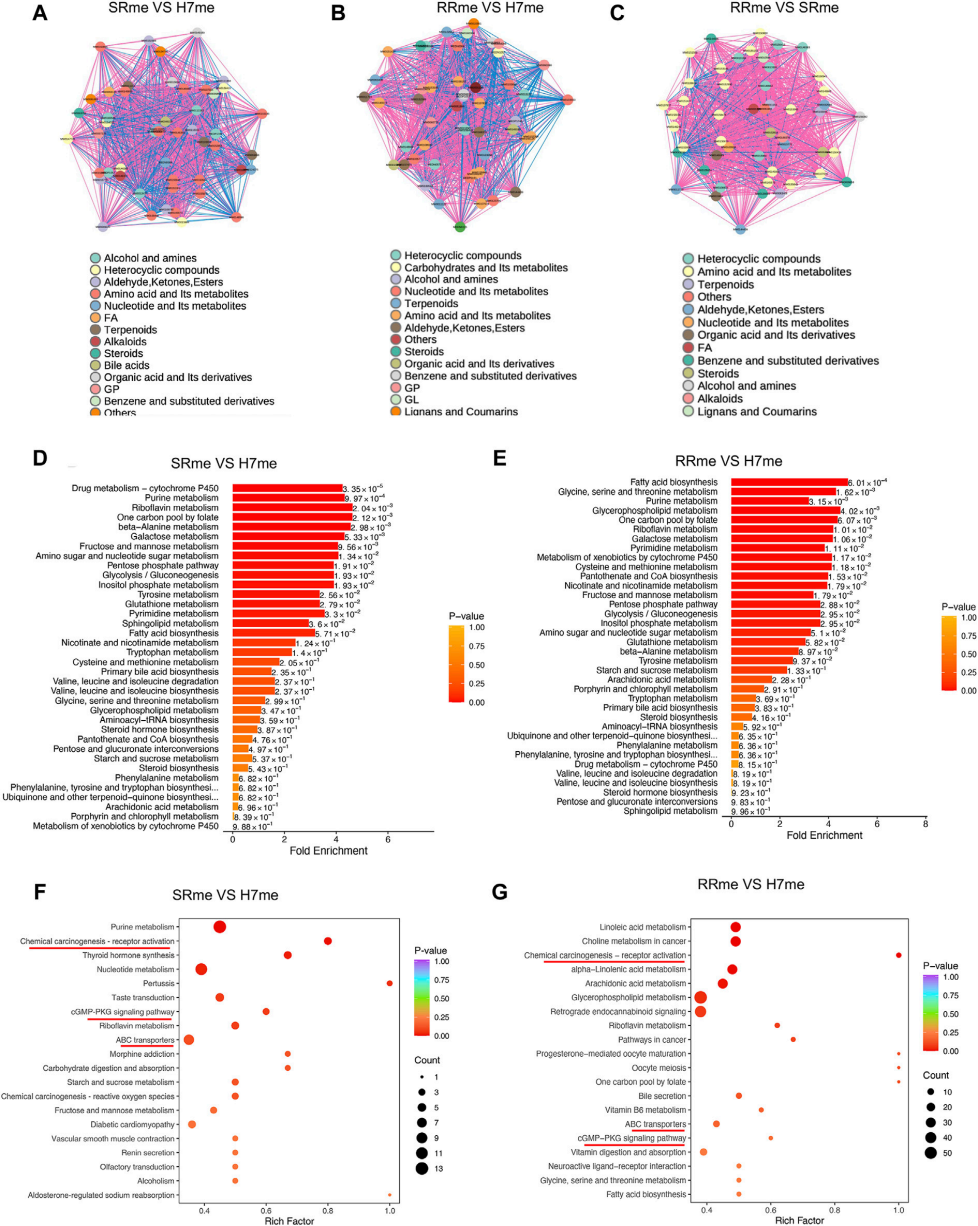
Functional enrichment analysis of differential metabolites. **(A–C)** Diagram portraying the correlation network of differential metabolites. The pink lines denoted positive correlation, while the blue lines indicated negative correlation. The line thickness was indicative of the absolute value of the correlation coefficient, with thicker lines denoting greater correlation strength. **(D,E)** Analysis of Metabolic Enrichment (MSEA). **(F,G)** Differential metabolite KEGG enrichment map. The horizontal axis corresponds to the Rich Factor associated with each pathway. The color of the point reflected the *p*-value, with redder points indicating greater significance of enrichment. The point size was proportional to the number of enriched differential metabolites. *n* = 3.

To conduct in-depth analysis of differential metabolites, we conducted correlation analysis and enrichment analysis on differential metabolites. The correlation between differential metabolites indicated that amino acid and its metabolites were most significant among the three groups compared (Figures 5A–C). Metabolic Enrichment Analysis (MSEA) found that a large number of metabolic pathways were enriched, including: drug metabolism cytochrome P450, amino acid metabolism, fatty acid biosynthesis, pure metabolism, and glycolis/gluconeogenesis (Figures 5D, E). KEGG analysis of differential metabolites uncovered that the signal pathways jointly enriched in Huh7/SR and Huh7/RR cells involve the activation of chemical carcinogenesis receptors, the CGMP-PKG signaling pathway, and ABC transporters (Figures 5F, G).

### Combination analysis of proteomic and metabolomic profiles

**FIGURE 6:**
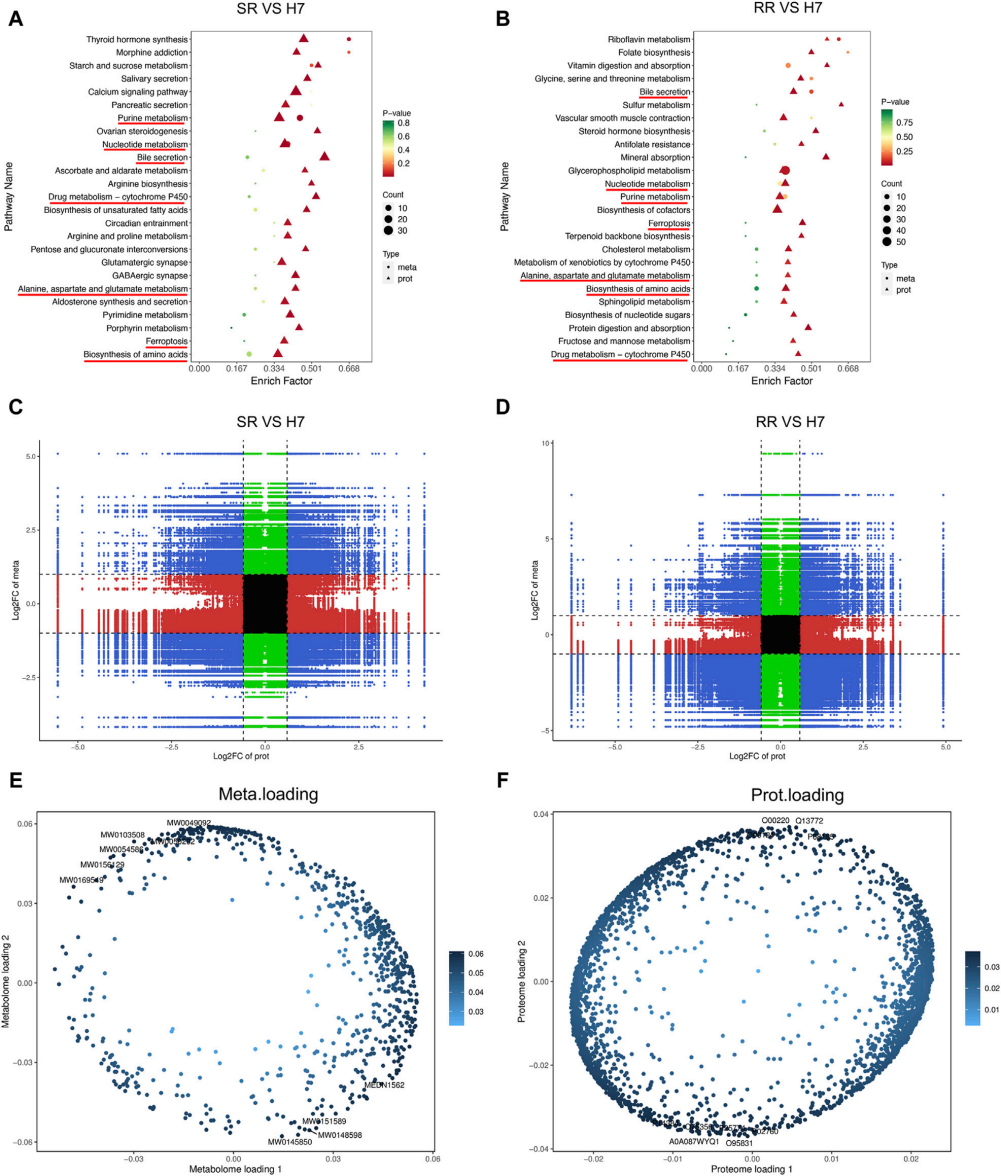
Combination analysis of proteomic and metabolomic profiles. **(A,B)** KEGG enrichment analysis bubble chart. The horizontal axis represented the enrichment factor (Diff/Background) of the pathway in different omics, while the vertical axis represents the name of the KEGG pathway. The gradient of red, yellow, and green showed a significant change in the degree of enrichment from high to medium to low, with *p*-value as the representative. The bubble shapes represented different omics, while the bubble sizes demonstrated the number of differential metabolites or proteins. The dots became larger as the numbers increase. **(C,D)** Correlation analysis nine quadrant chart. The horizontal axis represents the log_2_ FC of proteins, and the vertical axis represents the log_2_ FC of metabolites. **(E,F)** O2PLS analysis. *n* = 3.

By combining the proteomics and metabolomics analysis, it can be seen from the KEGG analysis results that the co-enriched signals of metabolism and proteomics in the two resistant cells are mainly reflected in: drug metabolism cytochrome P450, alanine, aspartate and glucose metabolism, ferroptosis, biosynthesis of amino acids, bill secretion, nucleotide metabolism, and pure metabolism (Figures 6A, B). The correlation analysis between proteins and metabolites revealed a intricate relationship within drug-resistant cells. Specifically, the third and seventh quadrants exhibited proteins and metabolites displaying a positive correlation, whereas the first and ninth quadrants indicated proteins and metabolites with discordant regulatory patterns (Figures 6C, D). Subsequently, we selected all proteins and metabolites that exhibited differential expression and utilized them to construct an O2PLS model. We then conducted a preliminary variable screening process where we identified variables that possessed a high correlation and weight in different data groups through load plots (Figures 6E, F). Through these analyses, we established a connection between differential metabolites and proteins, allowing for a comprehensive understanding of the underlying mechanisms of drug resistance.

### MISP, CHMP2B, IL-18, TMSB4X, and EFEMP1 are associated with drug resistance recurrence

**FIGURE 7:**
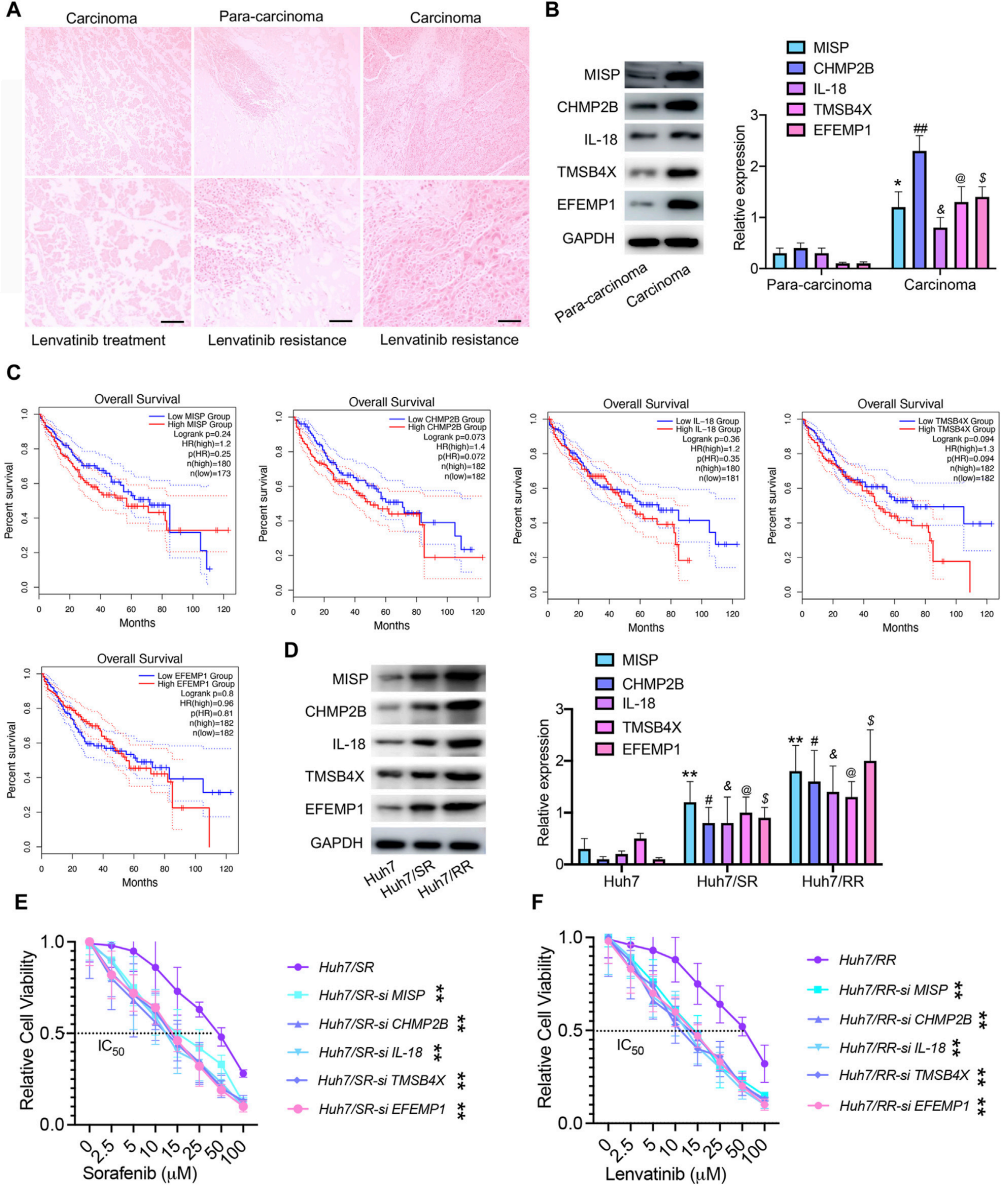
MISP, CHMP2B, IL-18, TMSB4X, and EFEMP1 are associated with drug resistance recurrence. **(A)** HE staining, Bar = 100 μm. **(B)** Immunoblotting was used to detect the expression levels of MISP, CHMP2B, IL-18, TMSB4X, and EFEMP1 proteins. Data are means ± SD from three experiments, analyzed by unpaired *t*-test: *p < 0.05; ^#^ *p* < 0.05; ^@^ *p* < 0.05; ^$^ *p* < 0.05; ^&^ *p* < 0.05, *n* = 3. **(C)** Overall survival of patients, grouped by high/low expression status of MISP, CHMP2B, IL-18, TMSB4X, and EFEMP1, plotted as Kaplan-Meier curve using the Gene Expression Profiling Interactive Analysis module (GEPIA). **(D)** Immunoblotting was used to detect the expression levels of MISP, CHMP2B, IL-18, TMSB4X, and EFEMP1 proteins. Data are means ± SD from three experiments, analyzed by One-Way ANOVA analysis: **p < 0.01; ^#^ *p* < 0.05; ^@^ *p* < 0.05; ^$^ *p* < 0.05; ^&^ *p* < 0.05, *n* = 3. **(E,F)** IC_50_ values of drug-resistant cells and drug-resistant cells transfected with si RNA. Data were analyzed by unpaired *t*-test: **p < 0.01, *n* = 3.

To validate the protein family spectrum findings in drug-resistant cells, we selected HCC tissues from patients who underwent lenvatinib treatment and those with recurrence after such treatment. HE staining revealed necrosis in HCC tissue from patients treated with lenvatinib, along with an enhanced presence of immune infiltrating cells in para-carcinoma tissues and a notable increase in cancer cells in recurrent carcinoma tissue. These observations corroborate our understanding of the proteomic profiles in drug-resistant HCC cells (Figure 7A). The expression of MISP, CHMP2B, IL-18, TMSB4X, and EFEMP1 proteins in carcinoma tissue was higher than that in para-carcinoma tissues (Figure 7B). Survival analysis showed that high expression of MISP, CHMP2B, IL-18, TMSB4X, and EFEMP1 is not associated with poor prognosis in HCC (Figure 7C). Therefore, we speculated that these 5 proteins may not be related to tumor growth, but rather to drug resistance. We found that the expression levels of these 5 proteins in drug-resistant cells were significantly higher than those in parental cells, both in sorafenib resistant cells and lenvatinib resistant cells (Figure 7D). In addition, we used siRNA technology to knock down the expression of these 5 proteins one by one, and after being knocked down, the IC50 values of drug-resistant cells significantly decreased (Figures 7E, F). These results suggest that targeting these proteins will reduce drug resistance.

## Discussion

Many HCC patients may develop drug resistance or relapse shortly after receiving first-line drug treatment, leading to poor treatment outcomes (Wang et al., 2024). The heterogeneity of HCC cells, their escape mechanisms, the existence of single nucleotide polymorphisms in drug metabolism, and the inadequate resilience of the patient’s immune system can all potentially contribute to the resistance of HCC patients to TKIs (Chen et al., 2022; Salani et al., 2022). Therefore, in-depth research on drug resistance mechanisms is essential for understanding the emergence of drug resistance in HCC cells. In our previous study, we discovered that knocking out XPO1 can effectively reduce the resistance of HCC cells to sorafenib (Wang et al., 2023b). The combination of XPO1 inhibitor KPT-8602 and sorafenib has a better tumor treatment effect than sorafenib alone. In this study, we established a comprehensive resistance spectrum, encompassing both metabolites and proteins. Notably, the co-enrichment signals observed in drug-resistant cells are primarily reflected in drug metabolism, specifically involving cytochrome P450 (Wei et al., 2022; McGill et al., 2023), amino acids and glucose metabolism (Guo et al., 2023b), ferroptosis (Guo et al., 2023a; Li et al., 2023), biosynthesis of amino acids, bill secretion, nucleotide metabolism, and pure metabolism. This also suggests that for the resistance mechanism of TKIs, we should not only focus on a single resistance target, but more research needs to be mapped to the overall resistance spectrum.

The protein and metabolic profile of drug-resistant cells is reshaped. We found that common differentially enriched signals in drug-resistant cells involve cell adhesion molecules. Previous studies have also shown that highly enriched in the processes of cell-cell adhesion response to sorafenib resistance (Chai et al., 2021). We also found that cell adhesion molecules are highly expressed in drug-resistant cells. Focal adhesion kinase (FAK) is a key factor in the resistance of lenvatinib in HCC (Hou et al., 2024). FAK inhibitor TAE226 combined with sorafenib reduces HCC growth *in vitro* and *in vivo* (Romito et al., 2021). In addition, we suggest that MISP, CHMP2B, IL-18, TMSB4X and EFEMP1 may serve as predictive biomarkers for TKIs treatment. Cultivating NK cells by activating IL-12 and IL-18 can promote the therapeutic effect of sorafenib (Eresen et al., 2024). Combined with the GEPIA database analysis, it was confirmed that the expression of these genes is not significantly correlated with the prognosis of HCC patients, which also suggests their potential important relationship with TKIs resistance. Drug-resistant cells exhibit reduced expression of IFITM3, CA4, AGR2, and SLC51B. Notably, SLC51B, a gene linked to liver metabolism and immune microenvironment (Cheng et al., 2021), suggests a pivotal role in the intricate relationship between TKIs resistance and liver metabolic immunity. Paradoxically, AGR2 is highly expressed in sorafenib resistant cells, supporting endoplasmic reticulum homeostasis and cell survival (Guo et al., 2016). This may be due to inconsistent drug concentrations used to establish drug-resistant cells. Additionally, we propose an important relationship between cellular metabolites and TKIs resistance. Drug metabolism cytochrome P450 (Naveed et al., 2021), amino acid metabolism, fatty acid biosynthesis, pure metabolism, and glycolis/gluconeogenesis are significantly enriched in drug-resistant cells. Sorafenib enhances cytochrome P450 lipid metabolites in patient with HCC (Leineweber et al., 2023), further underscoring the intricate link between cellular metabolism and drug resistance.

This study is not without limitations. While we detect the expression of MISP, CHMP2B, IL-18, TMSB4X, and EFEMP1 proteins in liver tissue samples from HCC patients receiving lenvatinib treatment, the same proteins were not detected in those receiving sorafenib treatment. This may be due, in part, to the fact that lenvatinib is currently the preferred drug in clinical practice. Nonetheless, we have further validated these results using drug-resistant cells and siRNA. In addition, the mechanisms of action between metabolic and protein profiles, as well as their relationship with drug resistance, require in-depth research.

In summary, targeting a single drug resistance mechanism is insufficient. A comprehensive approach combining protein and metabolomics interventions is crucial for reducing drug resistance in HCC from a holistic perspective.

## Data Availability

The original contributions presented in the study are publicly available. This data can be found here: https://www.ebi.ac.uk/pride/archive/projects/PXD052321, with identifier PXD052321.
