# Supplementary figures and images for: Protein and metabolic profiles of tyrosine kinase inhibitors co-resistant liver cancer cells
Source: Front Pharmacol. 2024 May 21;15:1394241. doi: 10.3389/fphar.2024.1394241 (PMC11149701; doi:10.3389/fphar.2024.1394241)

Figure S1

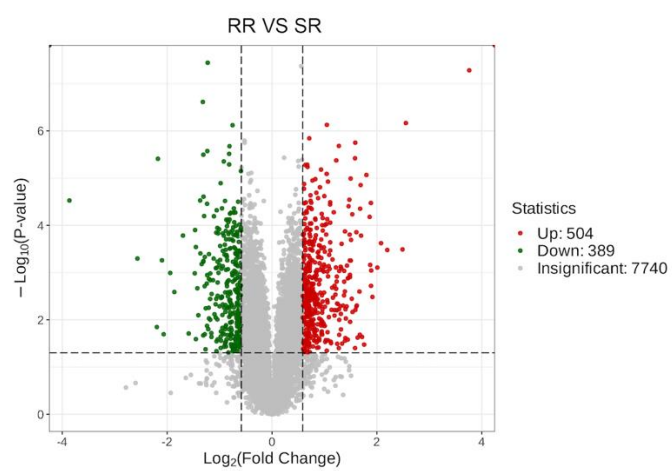

Figure S1. Differential protein volcano plot.

Supplement: Supplementary file 2 [file Image1.pdf]
